# Long-term outcomes of out-of-center veno-arterial ECMO cannulation for cardiopulmonary failure: investigation of prognostic parameters for a decision support tool – a 16-year retrospective study

**DOI:** 10.1186/s13049-025-01401-7

**Published:** 2025-05-12

**Authors:** Walter Petermichl, Alois Philipp, Maik Foltan, Andrea Stadlbauer, Peter-Paul Ellmauer, Christian Merten, Sebastian Blecha, Thomas Müller, Bernhard Ulm, Bernhard Graf, Dirk Lunz

**Affiliations:** 1 Department of Anesthesiology, University Medical Center Regensburg, Franz Josef Strauß Allee 11, 93053 Regensburg, Germany; 2 Department of Cardio Technology, University Medical Center Regensburg, Regensburg, Germany; 3 Department of Cardiothoracic Surgery, University Medical Center Regensburg, Regensburg, Germany; 4 Department of Cardiology, University Medical Center Regensburg, Regensburg, Germany; 5 Statistics Office, University Medical Center Rechts Der Isar, Munich, Germany

**Keywords:** Critical care interhospital transfer, Cardiopulmonary failure, Veno-arterial ECMO, Out of center, Indication criteria, Outcome, Cerebral performance category (CPC), ECOG performance status

## Abstract

**Background:**

Veno-arterial extracorporeal membrane oxygenation (VA ECMO) has served as a crucial intervention for critically ill patients with persistent cardiopulmonary failure. A standardized approach improves VA ECMO outcomes, which is why ECMO is currently limited to specialized centers. However, transferring critically ill patients to these ECMO centers is not without risk. Portable ECMO devices allow implantation in out-of-center settings prior to transportation. Despite efforts to standardize decision-making, significant variability remains, particularly in out-of-center (OoC) settings with limited data. Due to persistently high mortality, accurate indications are needed to optimize outcomes. This study aims to identify key factors associated with favorable outcomes in OoC VA ECMO and to develop practical decision-making tools for clinicians in these settings.

**Methods:**

We retrospectively investigated the outcomes of VA ECMO implantation in out-of-center settings between 2006 and 2022 at our institution. Parameters assessed prior to VA ECMO implantation, including organ failure count, mean arterial pressure (MAP), and laboratory data, were analyzed. Follow-up data were collected to evaluate functional (Eastern Cooperative Oncology Group [ECOG] performance status) and neurological (cerebral performance category score [CPC]) (outcomes. Statistical analyses were performed using non-parametric methods and SHAP importance analysis.

**Results:**

A total of 56.5% (195 of 345 patients) who underwent VA ECMO implantation in OoC survived, and 43.8% had a favorable neurological outcome (CPC 1). 37.6% of patients had good functional outcomes (ECOG 0–1). Patients with a MAP > 54 mmHg had better long-term functional outcomes, and those with a MAP > 64 mmHg had better mid-term neurological outcomes. Poor outcomes were associated with reduced coagulation activity and increased thrombogenicity. Renal and multi-organ failure prior to VA ECMO implantation were associated with poor neurological and functional outcomes.

**Conclusions:**

Through importance analyses, we identified key and secondary factors associated with favorable outcomes in OoC VA ECMO. The extent and severity of organ failure prior to VA ECMO implantation are crucial in determining outcomes. Hemodynamic status, as reflected by MAP, along with organ failure prior to VA-ECMO implantation, significantly influences neurological and functional outcomes. Patients with better hemodynamic stability and coagulation profiles had significantly improved chances of survival with favorable neurological and functional outcomes.

**Supplementary Information:**

The online version contains supplementary material available at 10.1186/s13049-025-01401-7.

## Background

Since the first successful application of the heart–lung machine in 1953, extracorporeal technology has continually evolved [1]. The use of veno-arterial extracorporeal membrane oxygenation (VA ECMO) has significantly expanded beyond the management of post-cardiotomy failure [2, 3]. VA ECMO has now become an established intervention for ensuring adequate tissue oxygenation and perfusion in critically ill patients who remain unstable despite optimal conservative management [4]. According to current scientific consensus, ECMO therapy should only be performed at specialized ECMO centers. This limitation arises from the current state of scientific knowledge and the associated guidelines for extracorporeal circulation [5, 6]. The CESAR trial demonstrated that an established infrastructure, coupled with standardized treatment strategies implemented by a highly experienced clinical team, is essential for improving outcomes in ECMO patients [5]. Due to these recommendations, patients must be transported to specialized ECMO centers. Critical care patients with a severe cardiopulmonary failure are at high risk during interhospital transfer because of this pre-transfer ECMO implementation may be indicated [7, 8]. Technological innovations and the development of smaller portable ECMO devices now enable implantation of ECMO prior to transport in out-of-center (OoC) settings [9].

Even if ECMO therapy is performed, the mortality rate remains high in critical care patients with a severe cardiocirculatory failure [10]. Considering the significant infrastructural requirements and the limited availability of ECMO therapy, providing the correct indication is essential for achieving favorable outcomes in these patients [11–13]. Even for patients within ECMO centers, where extensive information is available, determining the indication for extracorporeal therapy is not always straightforward [14]. The decision to initiate extracorporeal therapy for inter-hospital transfer in an OoC setting is even more challenging, as clinicians often only have access to limited data and must make decisions within a very short timeframe. Despite ongoing efforts by professional societies to standardize the decision-making process for ECMO indication, significant inconsistency and variability remain based on the individual physician and the specific ECMO patient [14]. To address this issue, research has commenced to identify factors influencing patient outcomes in extracorporeal therapy [15, 16]. Although colleagues have made significant efforts, questions regarding ECMO outcomes for our daily practice remain unresolved. As many of these ECMO outcome studies focus on a limited and often disparate set of outcome variables, it remains unclear whether specific parameters within these variables hold greater significance for the outcomes than others [8, 10, 17]. Only a limited number of studies examine patient outcomes for ECMO systems implanted OoC. However, OoC outcome studies frequently include patients treated with veno venous ECMO. In contrast, investigations of OoC VA ECMO therapies are significantly underrepresented [8]. Additionally, favorable neurological or functional long-term outcomes have been severely underrepresented as endpoints in existing studies compared to overall mortality [8, 16].

The aim of this study is to identify parameters associated with favorable neurological and functional long-term outcome following OoC VA ECMO therapy, based on the patient population at our ECMO center. This investigation will focus on variables routinely assessed in OoC settings to develop practical decision-making tools for ECMO indication. Furthermore, the study aims to assign weights to individual parameters to facilitate a decision-making process that extends beyond the simple criteria for indication.

## Material and methods

### Ethical approval

This study was approved by our local institution’s ethics committee (ethics committee file number: 19–121.56). Informed consent for the analysis of demographic, physiological, and hospital outcome data was not obtained, as this observational study did not alter any existing diagnostic or therapeutic strategies.

The procedures followed were in accordance with the ethical standards of the responsible committee on human experimentation (institutional or regional) and with the Helsinki Declaration of 1975.

### Study design

A retrospective review of our Regensburg ECMO database to identify critically ill patients with VA ECMO implanted in an OoC setting between January 2006 and November 2022. Demographic and clinical information were retrospectively extracted from our clinical database system. All names and identifying patient numbers were removed prior to analysis. Due to the retrospective nature of the study, clinicians were not blinded to the measurements. All patients included in the study primarily required VA ECMO.

### University medical center Regensburg OoC-ECMO-program

At the University Medical Center Regensburg, initial contact for OoC ECMO requests is established through a 24/7 emergency hotline. All relevant patient data were documented in a standard query log. Following the four-eye principle, indication for ECMO implantation was verified by an experienced medical team in accordance with current guidelines, based on the recommendations from the extracorporeal life support organization (ELSO).

### Implantation management in OoC-ECMO-therapy

At the University Medical Center Regensburg, OoC ECMO implantation is conducted by a specially trained ECMO team, consisting of a perfusionist and a specialist in anesthesiology and intensive care medicine. Depending on the distance to the hospital, the team is transported via ground or air. Upon the patient's arrival at the OoC facility, the team reevaluates the decision to implant the VA ECMO system based on actual laboratory results, respiratory assessments, and echocardiography. When feasible, consent is obtained from the patient or their family members.

Prior to the implantation procedure, the patients'pre-existing analgosedation was intensified. Vascular access for VA ECMO was achieved using percutaneous dilatational cannulation of the femoral vein and artery, a standard technique well-documented in the literature [3, 18–20]. We utilized a commercial extracorporeal life support (ECLS) cannulation set from Baummedical (47,495 Rheinberg, Germany) along with cannulas (HLS cannula, outflow 38/55 cm, 21/23 Fr; inflow 15 cm, 17/19 Fr, Getinge GmbH, 76,437 Rastatt, Germany). The CARDIOHELP® ECMO system (combined pump/oxygenator) from Getinge GmbH (76,437 Rastatt, Germany) was primarily employed. The perfusion of the lower extremity was evaluated before and after ECMO implantation using near-infrared spectroscopy (INVOS) and Doppler ultrasound.

In case of impaired leg perfusion, distal antegrade perfusion was initiated [21]. We thus implanted a specialized wire-reinforced perfusion cannula (DPC, CruraSave Femoral Perfusion Set 7 Fr, Free Life Medical GmbH, 52,080 Aachen, Germany) into the superficial femoral artery. All materials used in the ECMO circuit are commercially available and accredited.

From the time of implantation, continuous INVOS monitoring was performed on the lower extremities and bifrontally on the skull to identify and address any signs of hypoperfusion or oxygenation disturbances early [22]. After successful VA ECMO implantation the mean arterial pressure (MAP), was maintained at 55—70 mmHg. During ECMO support, we targeted an activated partial thromboplastin time of 55 ± 5 s, in line with current recommendations [23]. Additional details regarding anticoagulation protocols have been published previously [24].

Following implantation and cardiopulmonary stabilization, ventilator support was adjusted and optimized through blood gas analysis conducted pre- and post-oxygenator, as well as via the patient’s arterial line. For all etiologies (cardiac/non cardiac) guideline-compliant therapies were also administered.

Following successful treatment of the underlying condition, VA ECMO weaning was conducted according to center-specific criteria and standards, as well as current scientific guidelines [25]. Decannulation was performed either percutaneously with manual compression or a specialized vessel closure device (MANTA, Teleflex, 70,734 Fellbach, Germany) at the bedside or by surgeons in the operating theater after discontinuation of anticoagulation for at least 4 h.

### Follow-up examination of the outcome

We tracked patients after consent was obtained using telephone numbers and addresses provided by the patients or their families at hospital discharge. The first cerebral performance category score (CPC) was assessed at hospital discharge, and the ECOG was determined for the first time 3 months after discharge or transfer to a facility for continued care in survivors. Patients were considered lost to follow-up if we were unable to confirm their survival status after multiple attempts to reach them by phone or email. All survivors were invited for routine follow-up assessments, which included performance status (Eastern Cooperative Oncology Group, ECOG), neurological function (CPC), and evaluation of daily restrictions*.*

### Measurement and parameter selection

The laboratory values (lactate, prothrombin time, platelet count, etc.) were performed prior to VA ECMO implantation, out-of-center, according to the protocols of the referring hospitals. However, since all testing procedures were conducted using standardized, clinically validated, and calibrated laboratory analyzers, it is generally assumed that the measurement accuracy is comparable. Sample testing with the admission laboratory during ECMO therapy showed comparable results in patients. Due to the retrospective nature of the data analysis spanning over 16 years, an exact manufacturer specification, as is typical in a retrospective single-center or prospective multi-center study, could not be provided by the authors.

For the selection of clinical chemistry parameters, we focused on those routinely measured in critically ill patients and available even in non-tertiary care hospitals or university clinics. The goal of this study was to generate a valid prognostic estimate based on already existing parameters, aimed at supporting rapid decision-making. Additional easily accessible hemodynamic parameters included the number of organ failures, the SOFA score, central venous oxygen saturation, MAP, and catecholamine dosage. If no central venous saturation was available prior to ECMO cannulation (e.g., due to the absence of a central venous catheter), the central venous saturation (CVS) was measured from the venous drainage cannula before the initiation of extracorporeal circulation and was considered equivalent to the central venous saturation.

### CPC Scale – mid-term outcome measuring tool

The CPC score is a widely utilized metric in research and quality assurance for assessing neurological outcomes [19]. Patients were classified into one of two outcome groups: those who survived with a favorable neurological outcome (CPC score of 1) and those with neurological impairment or non-survivors (CPC scores 2, 3, 4, or 5). Although in some studies CPC 1 and CPC 2 are considered good neurological outcomes, the CPC 2 level is defined as moderate cerebral impairment. This allows for a broad interpretation of what constitutes"moderate". To achieve better discriminatory power, we decided in this study to classify only CPC 1 as a favorable neurological outcome [26]. In most cases, the CPC was determined multiple times, at least once at the time of hospital discharge or transfer to a facility for continued care. We defined the CPC status of our patients at the time of discharge from our center as a mid-term outcome. In cases where multiple CPC assessments were conducted, only the most recently documented CPC score was used in this study.

### Eastern Cooperative Oncology Group (ECOG) performance scale—long-term outcome measuring tool

First described in 1982 the ECOG performance status assesses the physical condition of cancer patients and quantifies overall well-being and limitations in activities of daily living [27]. The index ranges from 0 to 5, where 0 indicates unrestricted activity, as before the illness and 5 denotes death. The ECOG performance status provides a comprehensive overview of a patient's quality of life, allowing for illness-specific values to be determined for ECMO patients. In this study, we evaluated the ECOG score multiple times, first time 3 months after discharge or transfer to a facility for continued care for survivors or non-survivors, at the end of therapy. Since the ECOG score was re-evaluated annually whenever possible, we defined the ECOG status of the patients in this study as a long-term outcome criterion. Similar to the CPC, to achieve better discriminatory power, we decided in this study to classify ECOG 0 and 1 as indicative of a favorable neurological outcome. In cases where multiple ECOG assessments were conducted, only the most recently documented ECOG score for each patient was used in this study.

### Statistical analysis

All statistical analyses in this study were performed by an independent statistic office: Independent statistical consulting Bernhard Ulm, Munich. Continuous variables are presented using median with the interquartile range (IQR: 25 th/75 th percentiles), categorical variables are described by absolute and relative frequencies. Difference between the endpoints and influencing variables was tested using Mann–Whitney U tests for continuous variables and Chi^2^ or fisher's exact test was used. Due to missing values and multicollinearity classification was performed using Chi-squared Automatic Interaction Detection trees (CHAID). Variable importance of these trees was calculated using the model-agnostic approach and presented using variable importance plots. To assess the importance of the predictors in the CHAID decision trees, we used SHapley Additive ExPlanations (SHAP). This method describes the contribution of the individual variables to the predictions of the decision trees by calculating Shapley values. SHapley Additive exPlanations (SHAP) is a game-theoretic approach that assigns each feature a contribution value to the prediction of the model, thus ensuring a fair and consistent explanation of the importance of the variables. A significance level of *p* < 0.05 was set. All analysis were performed using IBM SPSS Statistics (Version 29) and R Version 4.4.2.

## Results

**Fig. 1:**
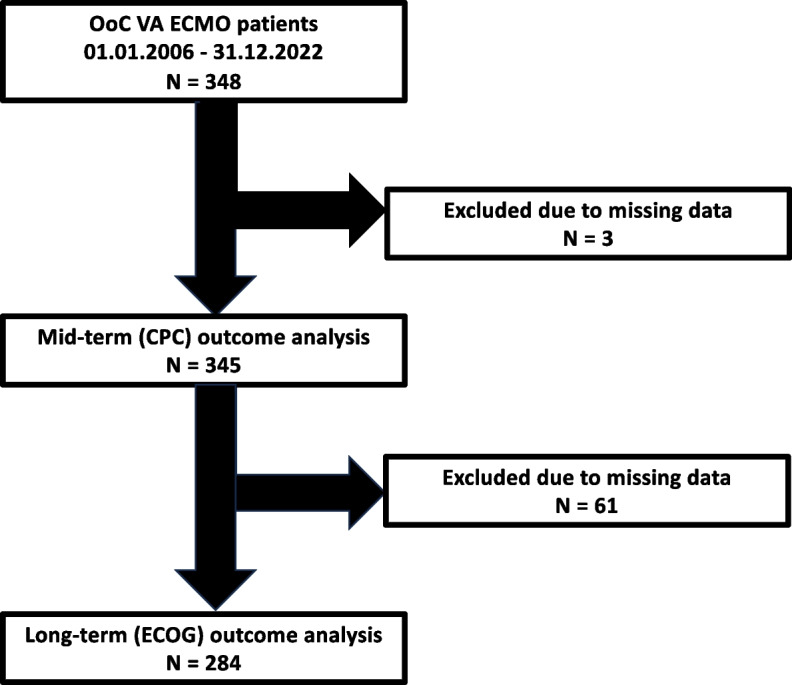
Flowchart of patient selection. Flowchart of exclusion criteria for OoC VA ECMO patients from 2006 to 2022

Between January 2006 and November 2022, a total of 1,402 VA ECMO procedures were performed at the University Hospital Regensburg. Of these, 348 procedures, representing approximately 24.8%, were percutaneous VA ECMO implantations conducted in an out-of-center setting. These patients were subsequently transported to the university hospital via intensive care transport vehicles or helicopters for further diagnostics and treatment. Thus, approximately one in four VA ECMO procedures at this institution was performed in an out-of-center setting, and the patients were later transferred for definitive care. Three of these patients had incomplete datasets, which prevented meaningful analysis; therefore, they were excluded from the study. A CPC score could be determined for all 345 patients included in the analysis. However, 61 patients were excluded from the follow-up analysis due to missing ECOG performance scale scores, resulting in a final cohort of 284 patients. The included and excluded patients of our study population are depicted in Fig. 1.

### Demographic data

Among the 345 patients analyzed, 239 (69.3%) were male and 106 (30.7%) female. The median age of all patients was 57 years (48 to 64 years). The average height was 170 cm (168 cm to 179 cm), and the average body weight was 80 kg (range: 70 kg to 95 kg). The median body mass index (BMI) was 27 kg/m^2^ (24 kg/m^2^ to 30 kg/m^2^). On average, patients were transported 70 km (range: 45 km to 102 km) following ECMO implantation.

The group with a favorable neurological outcome (CPC 1) had a significantly lower average BMI of 26 kg/m^2^ (24 kg/m^2^ to 29 kg/m^2^) compared to the group with a neurological outcome ≥ CPC 2, which had a BMI of 28 kg/m^2^ (24 kg/m^2^ to 31 kg/m^2^) (*p* = 0.048). Additionally, the average height of patients with a favorable neurological outcome was significantly taller, measuring 173 cm (170 cm to 180 cm), compared to those with poor neurological outcomes, whose average height was 170 cm (167 cm to 175 cm) (*p* = 0.009). No other demographic parameters showed significant differences between the groups with favorable and poor neurological outcomes (see Table 1).

**Table 1:** Demographic data in cerebral performance category (CPC) outcome group

	**Overall**	**CPC 1**	**CPC 2 to 5**	***p*-value**
	*N* = 345	*N* = 151	*N* = 194	
**Gender**				
**Male**	239 (69.3%)	107 (70.9%)	132 (68.0%)	0.6
**Female**	106 (30.7%)	44 (29.1%)	62 (32.0%)	
**Age(years)**	57 (48, 64)	56 (48, 64)	58 (48, 65)	0.3
**Height (cm)**	170 (168, 179)	173 (170, 180)	170 (167, 175)	0.009*
**Weight (kg)**	80 (70, 95)	80 (70, 90)	80 (70, 95)	0.4
**BMI (kg/m**^**2**^**)**	27 (24, 30)	26 (24, 29)	28 (24, 31)	0.048*

Demographic data for CPC in out-of-center VA ECMO patients between 2006 and 2022. The medians (25 th percentile/75 th percentile) are reported. The significance level was set at *p* ≤ 0.05. The significance level was set at *p* ≤ 0.05, and significant values are marked with an asterisk (*) in the table

When examining the demographic parameters in relation to functional outcomes after OoC VA ECMO, no significant differences were found based on the ECOG performance scale (see Table 2).

**Table 2:** Demographic data in ECOG performance scale outcome group

	**Overall**	**ECOG 0, 1**	**ECOG ** $\ge$** 2**	*p***-value**
	*N* = 284	*N* = 106	*N* = 178	
**Gender**				0.9
**Male**	194 (68.3%)	73 (68.9%)	121 (68.0%)	
**Female**	90 (31.7%)	33 (31.1%)	57 (32.0%)	
**Age (years)**	57 (48, 65)	57 (47, 64)	57 (49, 65)	0.3
**Height (cm)**	170 (167, 178)	172 (168, 180)	170 (166, 176)	0.065
**Weight (kg)**	80 (70, 95)	80 (74, 90)	80 (70, 95)	0.8
**BMI (kg/m**^**2**^**)**	27 (24, 30)	27 (25, 29)	27 (24, 31)	0.5

Demographic data for ECOG in out-of-center VA ECMO patients between 2006 and 2022. The medians (25 th percentile/75 th percentile) are reported. The significance level was set at *p* ≤ 0.05. The significance level was set at *p* ≤ 0.05, and significant values are marked with an asterisk (*) in the table

The median ECMO therapy duration for OoC VA ECMO patients was 5 days. However, individual patients exhibited considerable variation in therapy duration (ranging from 1 to 56 days). No significant difference in therapy duration was observed between the outcome groups, neither for CPC nor for ECOG (*p* > 0.9) The distribution of the underlying causes of cardiopulmonary failure at the time of OoC VA ECMO implantation can be found in (Table 3).

**Table 3:** Cause of cardiopulmonary failure in out-of-center VA ECMO

	**Overall ***N*** = 345**
Other cardiocirculatory failure (myocarditis, hypoxia, intoxication, hypothermia, hemorrhage)	**34** (9.9%)
Septic cardiomyopathy	**53** (15.4%)
Decompensated chronic heart failure	**49** (14.2%)
Acute myocardial infarction	**151** (43.8%)
Postoperative heart failure (non cardiac surgery)	**2** (0.6%)
Acute aortic dissection Typ A Stanford	**2** (0.6%)
Rhythmogenic heart failure	**13** (3.8%)
Postcardiotomy heart failure	**8** (2.3%)
Pulmonary embolism	**33** (9.6%)

Classification of patients according to underlying cause of cardiopulmonary failure, with patient count and percentage of the total population. Entries depict the number of patients (percentage)

After the failure of maximal conservative therapy, the indication for OoC VA ECMO therapy was made in case of persistent severe cardiopulmonary dysfunction. The indication for ECMO was classified into five groups according to the coding system used at our ECMO center: ECMO for low cardiac output without prior resuscitation (LCO), low cardiac output after successful resuscitation with return of spontaneous circulation (ROSC) (LCO-preCPR), low cardiac output with ongoing cardiopulmonary resuscitation (LCO-ongCPR), peri-interventional cardiopulmonary resuscitation (LCO-interCPR), and severe post-cardiotomy failure after cardiopulmonary bypass (LCO-postECB). The respective proportion of OoC VA ECMO patients in each category is shown in Table 4.

**Table 4:** Regensburger indication code for OoC VA ECMO

	Mid-term outcome (CPC)	Long-term outcome (ECOG)
	*N* = 345	*N* = 284
LCO-ongCPR	82 (23.8%)	64 (22.5%)
LCO-interCPR	21 (6.1%)	17 (6.0%)
LCO	104 (30.1%)	88 (31.0%)
LCO-preCPR	136 (39.4%)	113 (39.8%)
LOC-postECB	2 (0.6%)	2 (0.7%)

OoC VA ECMO indications according to the coding system used at the University Hospital Regensburg. Entries depict the number of patients (percentage)

For both outcome groups (CPC and ECOG), it was observed that despite the exclusion of 61 patients in the ECOG outcome group (due to missing follow-up data), the percentage distribution of the indication groups remained consistent.

### CPC score and ECOG performance scale outcome in OoC VA ECMO

Assessed according to the cerebral performance category score, the following mid-term outcome distribution was observed among the out-of-center VA ECMO patients (see Table 5).

**Table 5:** Cerebral performance category (CPC) outcome in Out-of-Center VA ECMO

Cerebral performance category	overall *N* = 345
CPC 1	**151** (43.8%)
CPC 2	**39** (11.3%)
CPC 3	**4** (1.2%)
CPC 4	**1** (0.3%)
CPC 5/non survivor	**150** (43.4%)

Outcome distribution into the individual CPC groups in the OoC VA ECMO population; entries represent the number of patients (percentage)

Based on the collected data, it could be shown that the OoC VA ECMO program at the University Hospital Regensburg achieved a mid-term survival rate of 57.6%. Survival with a good neurological outcome (CPC 1) was 43.8%, while survival with a good neurological outcome (CPC 1) and neurological outcome defined as lower limitations in daily functioning (CPC 2) was observed in 55.1% of cases.

Among the 284 patients (61 of 345 patients were missing in the follow-up) who were assessed using the ECOG performance scale, the following outcome distribution was observed (see Table 6).

**Table 6:** Fate of patients after Out-of-Center VA ECMO

	**overall ***N*** = 345**
Patients who died during ECMO Therapy	**150** (42,5%)
Survivors with:	195 (57.6%)
- Recovery after Conservative Therapy or Percutaneous Transluminal Coronary Angioplasty (PTCA)	129 (66.2%) *
- Recovery after Coronary Artery Bypass Grafting (CABG)	13 (6.7%) *
- Recovery after Valve Replacement or Valvuloplasty	11 (5.6%) *
- Recovery after Aortic Replacement	1 (0.5%) *
- Recovery after Repair of a Ventricular Septal Defect (VSD)	1 (0.5%) *
- Left Ventricular Assist Device (LVAD)	13 (7.7%) *
- Heart Transplantation	11 (6.2%) *
- Recovery following Other operative Therapies (Embolectomy, Pneumonectomy, etc.)	13 (6.5%) *

Fate of Patients After Out-of-Center VA ECMO entries represent the number of patients (percentage and percentage of survivors marked with asterisk (*))

Based on the collected data, it could be shown that the OoC VA ECMO program at the University Hospital Regensburg achieved a long-term survival rate of 48.5%. Survival with a good functional outcome (ECOG 0,1) was 37.6%, while survival with an acceptable functional outcome (ECOG 1,2,3) — defined as more than 50% bedridden per day — was observed in 47.1% of cases.

The median survival after OoC VA ECMO cannulation was determined to be 679 days (328 days; 5065 days) in the follow-up assessments of this study. This corresponds to an average of 1.9 years (0.9 years; 13.9 years) of life gained. Patients who died during therapy before hospital discharge had a median survival of 5 days (1 day; 109 days) after initiation of VA ECMO. For better comparability (possible 10-year follow-up) with other studies, only patients who were cannulated for OoC VA ECMO between 2006 and 2012 (*N* = 42) were considered. In this subset of our study population, a survival rate of 42.6% with good neurological outcome (CPC 1) was observed, which is comparable to the overall study population (2006 to 2022). The median survival in this subgroup was 3830 days (466 days; 5895 days), corresponding to 10.5 years (1.3 years; 16.2 years).

### Intracranial pathologies as a factor influencing neurological outcome in CPC and ECOG scales

A major cause of persistent neurological or functional impairment is acute intracranial pathology. For this reason, we investigated our patient cohort for the presence of strokes, intracranial hemorrhages, and brain tumors. In the total cohort of 348 patients, we identified 41 cases of stroke, two cases of intracerebral hemorrhage, two cases of meningioma, and three cases of pre-existing traumatic brain injury. This corresponds to a rate of 13.2% among all OoC VA ECMO patients.

When correlated with the CPC outcome, we found that intracranial pathology was present in non-survivors in 20 cases. Eighteen patients with acute intracerebral pathology were in the CPC 1 group, and eight were in the CPC > 2 group. Interestingly, the correlation with the ECOG scale showed a different distribution. While the number of deaths remained the same, only eight of the 46 patients were classified as ECOG 0 or 1, while 17 patients were classified as ECOG > 2.

### Disease dynamic and organ failure prior to OoC VA ECMO

The analysis of the collected data revealed that pre-existing organ failure was associated with poor outcomes, both in the neurological (CPC) and functional (ECOG) outcome group. Specifically, renal failure or multi-organ failure involving more than two organs was highly significant in relation to poor outcomes. The SOFA score, originally developed to assess the severity of sepsis and evaluate the function of multiple organ systems, was also found to be a highly significant predictor of outcome in cases of multi-organ dysfunction due to cardiopulmonary failure (see Table 7).

**Table 7:** ECOG performance scale outcome in Out-of-Center VA ECMO

ECOG-performance scale,	overall *N *= 284
0 ECOG	**16** (5.6%)
1 ECOG	**90** (32%)
2 ECOG	**27** (9.5%)
3 ECOG	**4** (1.4%)
4 ECOG	**0** (0%)
5 ECOG = non survivors	**147** (51.5%)

Outcome distribution into the individual ECOG groups in the OoC VA ECMO population; entries represent the number of patients (percentage)

Surprisingly, prior resuscitation was not significant in distinguishing outcomes (*p* = 0.11 for CPC and *p* = 0.071 for ECOG). The two parameters chosen to assess disease dynamics — ventilation days prior to ECMO and time from onset of illness to ECMO therapy — were not significant for the ECOG group. a significant relationship was found for the time from illness onset to ECMO initiation in CPC. The distance to the implantation site was not significant in the CPC outcome analysis (*p* = 0.11), but in the ECOG outcome analysis, patients with good functional outcomes were, on average, treated at more distant centers with OoC VA ECMO.

### Classical hemodynamic parameters prior to OoC VA ECMO Mean arterial pressure (MAP), central venous saturation (ScvO₂), catecholamines

Regarding classical hemodynamic parameters, the MAP showed a highly significant correlation with outcome in both the CPC and ECOG group. In the CPC > 2 group, the MAP was significantly lower at 55 mmHg (45 mmHg; 66 mmHg) compared to the CPC 1 group, where it was 60 mmHg (55 mmHg; 67 mmHg) (*p* = 0.003). Similarly, in the ECOG 0, 1 group, the mean MAP was 60 mmHg (55 mmHg; 67 mmHg), significantly higher than in the ECOG > 2 group, where it was 55 mmHg (45 mmHg; 65 mmHg) (p < 0.001).

ScvO₂was significantly higher in the CPC 1 group at 54% (38%; 64%) compared to the CPC $\ge$ 2 group at 44% (26%; 59%) (*p* < 0.001). A similar trend was observed in the ECOG group, where ECOG 0 and 1 had a significantly higher saturation of 53% (37%; 64%) compared to ECOG $\ge$ 2, which was 46% (28%; 59%) (*p* = 0.020).

Among the catecholamines, only the dosage of epinephrine showed a significant difference in outcome. Higher epinephrine dosing of 0.5 µg/kg/min (0.0 µg/kg/min; 2.0 µg/kg/min) was significantly associated with a worse functional outcome (*p* = 0.014) and with a worse CPC outcome (*p* = 0.013). The doses of norepinephrine were not significantly different for either the CPC or the ECOG outcomes: CPC 1 was 0.64 µg/kg/min (0.37 µg/kg/min; 1.14 µg/kg/min) versus CPC $\ge$ 2 at 0.71 µg/kg/min (0.31 µg/kg/min; 1.20 µg/kg/min) (*p* = 0.7), and ECOG 0, 1 was 0.63 µg/kg/min (0.37 µg/kg/min; 1.14 µg/kg/min) versus ECOG $\ge$ 2 at 0.73 µg/kg/min (0.38 µg/kg/min; 1.23 µg/kg/min) (*p* = 0.3).

### Coagulation alterations prior to OoC VA ECMO

A significant difference in the release of fibrin degradation products (D-Dimer) as well as in the plasma coagulation parameter INR/Quick was observed in both outcome groups, CPC and ECOG (see Table 8). In both CPC and ECOG, the poorer outcome group exhibited increased consumption and/or reduced synthesis of plasmatic coagulation factors (INR/Quick). Regarding platelets, a significant decrease in platelet count was also noted in the CPC outcome group with poor neurological outcome (see Table 8). No significant difference in the partial thromboplastin time (PTT) was observed in either of the outcome analyses.

**Table 8:** Disease dynamic and organ failure prior to Out-of-CenterVA ECMO CPC outcome

	**Overall *N* = 345**	**CPC 1**	**CPC ≥ 2**	***p*-value**
**Resuscitation prior to ECMO**	227 (65.8%)	92 (60.9%)	135 (69.6%)	0.11
**Ventilation days prior to ECMO**	0 (0, 1)	0 (0, 1)	0 (0, 1)	> 0.9
**Time from onset of illness to ECMO in days**	0 (0, 1)	1 (0, 1)	0 (0, 1)	0.003*
**Dialysis prior to ECMO**	61 (17.7%)	16 (10.6%)	45 (23.2%)	0.002*
**SOFA-score prior to ECMO**	15 (13, 17)	14 (13, 17)	16 (14, 18)	< 0.001*
** ≥ 2 Organ failures prior to ECMO**	182 (52.8%)	63 (41.7%)	119 (61.3%)	< 0.001*
**Distance to implantation site in km**	70 (45, 104)	72 (50, 110)	70 (43, 101)	0.11
	**Overall *N* = 284**	**ECOG 0, 1**	**ECOG ≥ 2**	***p*-value**
**Resuscitation prior to ECMO**	186 (65.5%)	62 (58.5%)	124 (69.7%)	0.071
**Ventilation days prior to ECMO**	0 (0, 1)	0 (0, 1)	0 (0, 1)	0.8
**Time from onset of illness to ECMO in days**	1 (0, 2)	1 (0, 2)	1 (0, 2)	0.3
**Dialysis prior to ECMO**	50 (17.6%)	9 (8.5%)	41 (23.0%)	0.007*
**SOFA-score prior to ECMO**	15 (13, 18)	14 (12, 17)	16 (14, 18)	< 0.001*
** ≥ 2 Organ failures prior to ECMO**	150 (52.8%)	44 (41.5%)	106 (59.6%)	0.005*
**Distance to implantation site in km**	70 (45, 102)	80 (60, 110)	70 (40, 95)	0.022

Presentation of disease severity due to perfusion disturbance with evidence of organ failures. Specifically, renal failure and the SOFA score, commonly used in sepsis to assess organ failures, are highlighted. Disease dynamics are scaled by the duration of ventilation prior to ECMO, as well as the time from the onset of illness to the need for ECMO therapy. Values were presented in number of patients (percentage) and main (QRS values) (25 th and 75 th percentiles). The significance level was set at *p* ≤ 0.05, and significant values are marked with an asterisk (*) in the table

### Hemoglobin (Hb), lactate dehydrogenase (LDH), aspartate aminotransferase (ASAT), and bilirubin prior to OoC VA ECMO

In our study, no significant difference in hemoglobin concentration was observed between the outcome groups in patients undergoing OoC VA ECMO (*p* = 0.9 for CPC; *p* = 0.072 for ECOG). However, LDH levels demonstrated a significant difference in the subgroup analysis of patients with a MAP < 54 mmHg (*N* = 110), with a threshold of 456 U/L prior to OoC VA ECMO implantation. Patients with ECOG 0—1 had significantly lower LDH levels compared to those with ECOG ≥ 2 (*p* = 0.014). In the overall study population, LDH did not differ significantly between outcome groups (*p* = 0.2 for CPC; *p* = 0.2 for ECOG). Similarly, ASAT (*p* = 0.4 for CPC; *p* = 0.2 for ECOG) and total bilirubin levels (*p* = 0.3 for CPC; *p* = 0.5 for ECOG) prior to OoC VA ECMO implantation did not differ between patients with good versus poor outcomes.

### Systemic inflammatory response prior to ECMO implantation

In the analysis of serum inflammatory markers measured prior to the initiation of VA ECMO, no significant differences were found between outcome groups for C-reactive protein (CRP) (*p* = 0.6 for CPC; *p* = 0.5 for ECOG) and Interleukin—2 (IL—2) (*p* = 0.3 for CPC; *p* = 0.3 for ECOG). However, significant differences were observed for Interleukin—6 (IL—6) (*p* = 0.002 for CPC; *p* = 0.009 for ECOG) and Interleukin-8 (IL—8) (*p* = 0.005 for CPC; *p* = 0.007 for ECOG), with IL—6 and IL—8 levels being 1.5 to 2.5 times higher in patients with poor outcomes compared to those with favorable outcomes (see Table 9). In patients with a MAP between 54–64 mmHg and intact coagulation (*N* = 78), a rise in Tumor Necrosis Factor Alpha (TNF-α) greater than 19 pg/ml was identified as a significant prognostic marker for worse outcomes (*p* = 0.012). However, in the ECOG outcome group, TNF-α was not significantly different between patients with good and poor functional outcomes (*p* = 0.2 for ECOG) (Table 10).

**Table 9:** Coagulation parameters (platelets, PTT, Quick, INR, D-Dimer)

Overall *N* = 345	CPC 1	CPC ≥ 2	*p*-value
Platelet count (/µL)	217 (158, 292)	186 (125, 256)	0.011*
Quick (%)	61 (44, 86)	56 (37, 79)	0.078
INR	1.35 (1.10, 1.80)	1.50 (1.20, 2.10)	0.018*
PTT (sec)	55 (35, 88)	50 (36, 86)	> 0.9
D-Dimer (μg/mL)	7 (2, 21)	11 (4, 35)	0.003*
Overall *N* = 284	ECOG 0, 1	ECOG ≥ 2	*p*-value
Platelet count (µL)	215 (159, 280)	190 (125, 269)	0.13
Quick (%)	62 (44, 89)	53 (36, 74)	0.007*
INR	1.30 (1.10, 1.70)	1.50 (1.20, 2.10)	0.004*
PTT (sec)	56 (35, 86)	54 (36, 89)	0.8
D-Dimer (μg/mL)	7 (2, 19)	11 (4, 35)	0.003*

Comparison of cellular and plasma coagulation in OoC VA ECMO patients with good and poor outcomes. The upper part of the table presents CPC outcomes, while the lower part shows ECOG outcomes. Values were presented as main values and QRS values (25 th and 75 th percentiles). The significance level was set at *p* ≤ 0.05, and significant values are marked with an asterisk (*) in the table

**Table 10:** Inflammatory markers prior to Out-of-Center VAECMO

Overall *N* = 345	CPC 1	CPC ≥ 2	*p*-value	Available data prior to ECMO
CRP	20 (5, 136)	26 (4, 116)	0.6	276/345
TNF alpha	16 (11, 25)	18 (12, 29)	0.12	243/345
TNF alpha subgruppe (MAP 54–64 mmHg; Quick > 42%)	$\le$19	> 19	0.012	243/345
IL—2	1,048 (601, 2,062)	1,252 (597, 2,799)	0.3	247/345
IL—6	378 (112, 2,116)	909 (274, 4,494)	0.002*	256/345
IL—8	162 (58, 452)	263 (113, 727)	0.005*	251/345
Overall N = 284	ECOG 0, 1	ECGO ≥ 2	p-value	
CRP	18 (5, 140)	29 (5, 127)	0.5	276/345
TNF alpha	16 (11, 27)	18 (12, 31)	0.2	243/345
IL—2	1,012 (571, 2,608)	1,274 (635, 2,743)	0.3	247/345
IL—6	384 (93, 2,755)	960 (283, 4,494)	0.009*	256/345
IL—8	155 (55, 654)	273 (123, 903)	0.007*	256/345

Comparison of inflammatory markers in out-of-center (OoC) VA ECMO patients with good and poor outcomes. The upper part of the table presents CPC outcomes, while the lower part shows ECOG outcomes. Values were presented as main values and QRS values (25th and 75th percentiles). The significance level was set at *p* ≤ 0.05, with significant values marked by an asterisk (*) in the table

### SHAP Importance Analysis

**Fig. 2:**
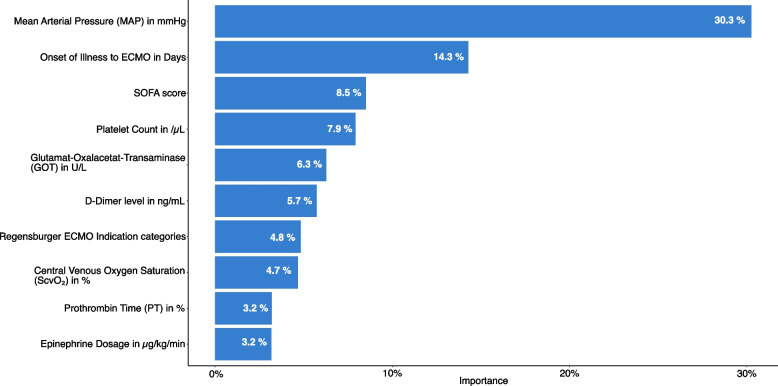
SHAP Importance: The size of variable influence on the CPC outcome in Out-of-Center VA ECMO patients. SHAP Importance: The size of the variable influence, sorted in descending order of importance, on the CPC outcome, expressed as a percentage

**Fig. 3:**
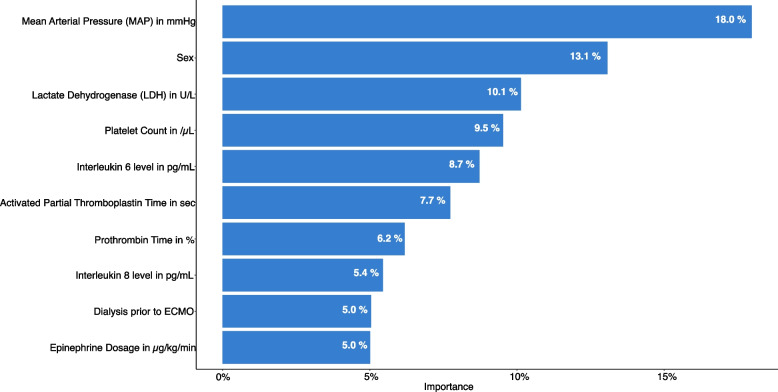
SHAP Importance: The size of variable influence on the ECOG outcome in Out-of-Center VA ECMO patients. SHAP Importance: The size of the variable influence, sorted in descending order of importance, on the ECOG outcome, expressed as a percentage

Since several potential influencing factors for neurological and functional outcomes were identified in the individual analyses, a SHAP (SHapley Additive exPlanations) Importance Analysis was performed to assess the contribution of each factor. Figure 2 illustrates the importance of each variable in relation to the CPC outcome, while Fig. 3 illustrates the importance in relation to the ECOG outcome.

We conducted a SHAP importance test for each node of the decision trees to identify the most influential variable. In Figs. 2 and 3, the SHAP analyses for the first nodes of the CPC and ECOG decision trees are shown. These analyses revealed that the most important factor for distinguishing between a good and poor outcome in OoC VA ECMO was the mean arterial pressure.

**Fig. 4:**
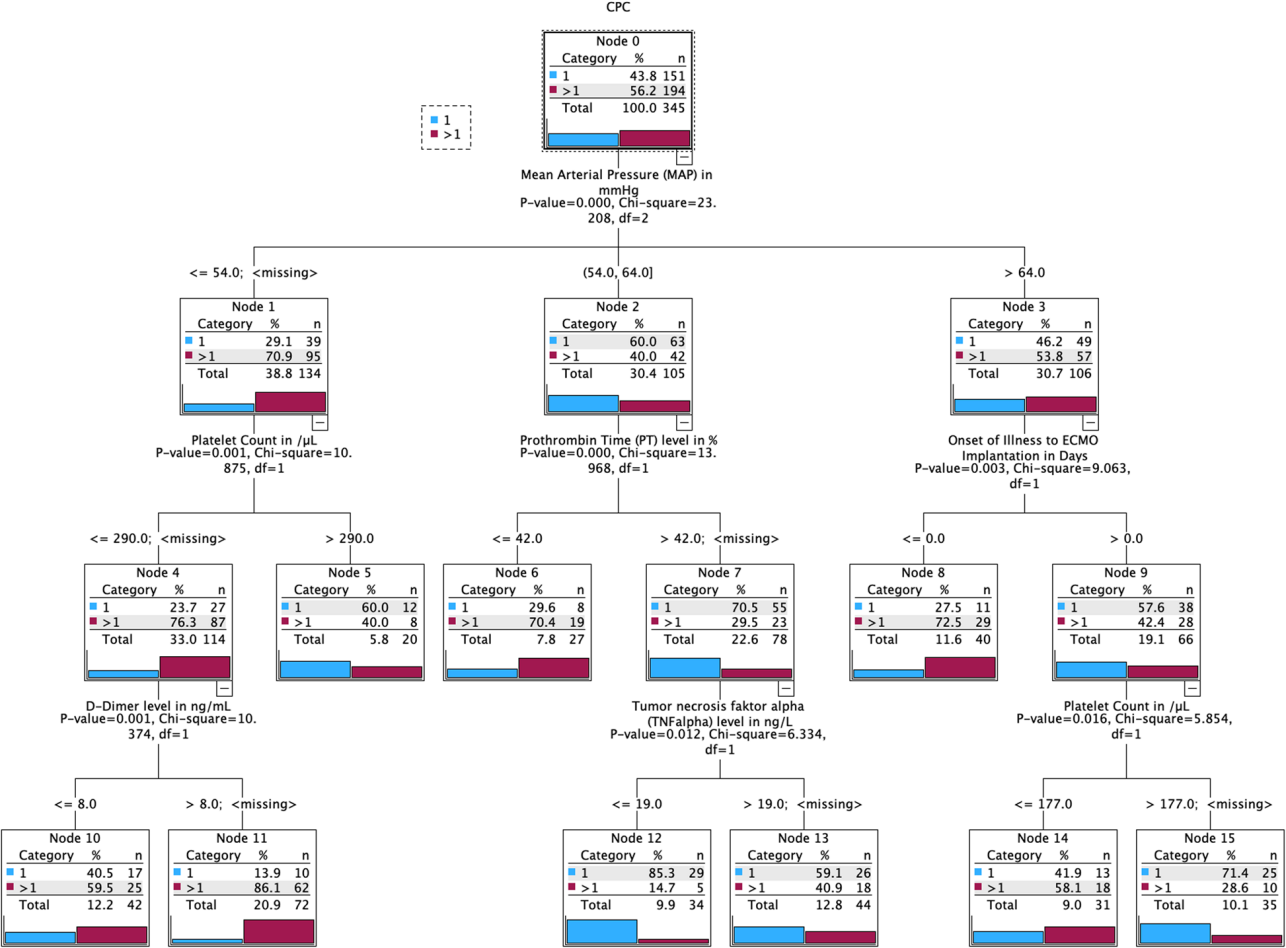
Decision tree mid-term outcome (CPC) in OoC VA ECMO. The outcome variables are presented in descending order of importance, organized in a decision tree format. For each variable (represented as nodes), the calculated threshold, sample size(N), percentage distribution (CPC group and total population), and p-value are provided. The significance level was set at *p* < 0.05

**Fig. 5:**
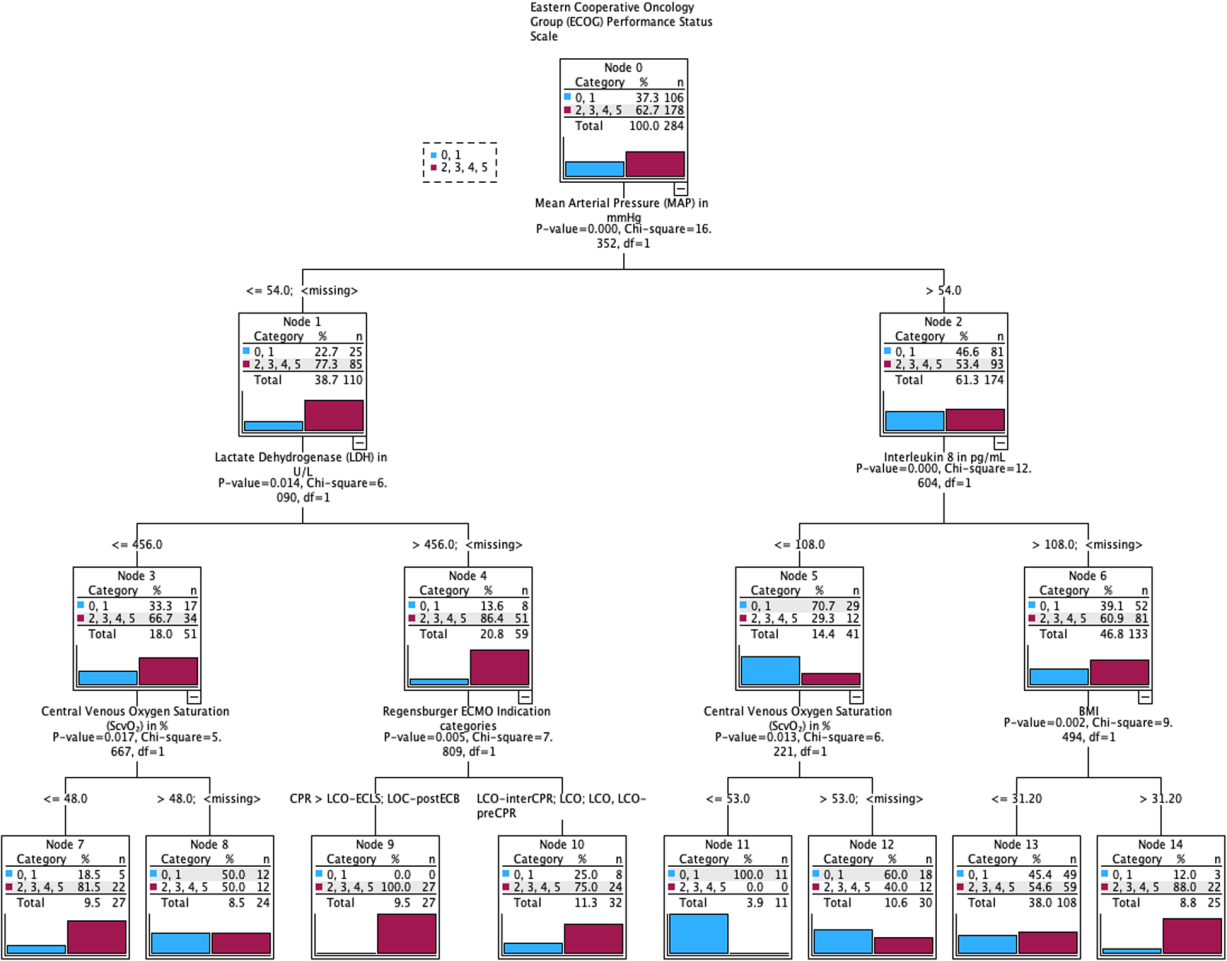
Decision tree long-term outcome (ECOG) in OoC VA ECMO. The outcome variables are presented in descending order of importance, organized in a decision tree format. For each variable (represented as nodes), the calculated threshold, sample size (N), percentage distribution (ECOG group and total population), and p-value are provided. The significance level was set at *p* < 0.05

For the subsequent nodes, a new SHAP analysis was performed for each, corresponding to the smaller cohort of patients. The nodes displayed in Figs. 4 and 5 represent the variables with the highest percentage in the SHAP analysis. The boxes within each decision node list the categories according to the respective percentage and the number of patients (N) in the corresponding variable group.

### Linked individual criteria for determining neurological outcomes in OoC VA ECMO

Based on the SHAP Importance ranking, a decision tree was created to enable the sequencing of multiple individual outcome criteria. Starting with the most influential factor, the MAP, subsequent variables were assigned, and a threshold for each influence factor was calculated to differentiate between good and poor outcomes. This analysis was performed separately for neurological outcomes, as measured by the CPC scale (Fig. 2), as well as for functional outcomes, as measured by the ECOG scale (Fig. 3).

Figure 4 shows that when the mean arterial pressure (MAP) is > 65 mmHg, the time from the onset to ECMO implantation predominantly influences the prognosis. The faster the cardiopulmonary failure occurs, the worse the outcome. This often reflects the resuscitation situation. A platelet count below 177/nL in this context is associated with a poor outcome. A MAP of 54–64 mmHg is associated with a poor prognosis when accompanied by a low prothrombin value. With a preserved prothrombin level, high inflammation, as measured by tumor necrosis factor-alpha, is associated with a poor outcome. In contrast, a MAP < 54 mmHg is associated with a poor outcome when the platelet count is < 299/nL. Among these patients, those with a low D-dimer load had the best chances.

In the ECOG group (shown in Fig. 5), the MAP value is the most important influencing factor for survival. Patients with an arterial mean pressure greater than 54 mmHg and a low Interleukin 8 level (< 108 pg/mL) more often had a good outcome. Patients with elevated Interleukin 8 levels had the best chance of a favorable prognosis when the Body Mass Index was < 31.2 kg/m^2^. In the group of patients with a MAP value less than 54 mmHg, those with a low LDH level (< 456 U/L) more frequently had a good outcome if the central venous saturation did not fall below 48%. Among patients with high LDH levels, the highest chance of a good outcome was observed when the cause of low cardiac output occurred peri-procedurally during PTCA or when the low cardiac output did not occur during or after ECMO.

## Discussion

### Outcome in OoC VA ECMO

Analysis of data from 2006 to 2022 on OoC VA ECMO at our ECMO center revealed a mid-term outcome with a good neurological outcome (CPC ≤ 1) in 43.8% of patients, and a long-term outcome with good functional status (ECOG ≤ 1) in 37.3% of patients. Direct comparison of our OoC VA ECMO outcome results with other VA ECMO studies is challenging for two main reasons. Firstly, our literature review revealed a limited number of studies addressing OoC VA ECMO, most of which were characterized by small sample sizes [28]. Secondly, when attempting to compare our OoC VA ECMO outcome results with VA ECMO studies that did not involve OoC implantation, we found that these studies often used mortality reduction as the primary outcome parameter [14, 29]. In a study by Combes et al. that examined the long-term functional outcomes of VA ECMO patients in cardiogenic shock, similar results were reported, despite a considerably smaller cohort [16]. In a larger study population by Cankar et al., which investigated long-term survival (10-year follow-up) after VA ECMO, the average survival time was 8.7 years [30]. In our study, we were able to demonstrate a similar, even slightly longer survival time of 10.5 years in the subgroup analysis for the 10-year follow-up. The survival rate in the study by Cankar et al. was significantly lower at 31.4% compared to other comparable publications, which reported survival rates of 40% ± 4% after VA ECMO with good neurological outcomes [16, 31, 32]. With a 47.6% survival rate, the 10-year follow-up subgroup was comparable to the survival rate of the overall study population (43.8%) and to other studies on survival after VA ECMO. The implantation of OoC VA ECMO does not appear to have a detrimental effect on survival rates or survival duration.

Since there is no standardized definition for good or poor outcomes, the outcome could potentially be influenced by the use of restrictive or liberal thresholds for the CPC/ECOG scores as cut-off points. For the cut-off point for the CPC score, we based our approach on ECLS publications that investigated neurological outcomes following intrahospital cardiac arrest (IHCA), out of hospital cardiac arrest (OHCA), and VAECMO [33, 34]. However, since a majority of the VA ECMOs in this study (242/345) were not implanted under resuscitation, we deemed it necessary to set a higher standard for the outcome. Therefore, a favorable neurological outcome was classified with a CPC of 1. Similarly, we defined a good functional outcome as an ECOG score of ≤ 1. Since the ECOG performance status (assessment of daily living abilities) could often not be reliably determined prior to hospital discharge, we used the cerebral performance category (CPC) score for outcome measurement, even though, in our view, it offers significantly lower discriminative power for the outcome [31].

### Outcome variables

Several studies have already investigated potential influencing variables on mortality or short- and long-term outcomes in VA ECMO patients. Notably, in most of these studies, there is considerable heterogeneity in the selection of variables [8, 17]. The literature shows a wide range of clinical scoring systems (e.g., SOFA score), catecholamine dosages, laboratory values (e.g., INR), and hemodynamic measurements (e.g., MAP) among the variables investigated [14]. One common feature of all these variables is that they appear to have a significant impact on VA ECMO outcomes, yet they were often studied in isolation as predictors of outcome. This study demonstrates that patient selection based on a single parameter does not adequately reflect reality. By linking multiple variables according to their importance, a decision tree was developed that more clearly identifies the distinguishing factors for outcomes. For example, patients with a MAP < 54 mmHg, normal platelet count, and low D-Dimer levels have a real chance of achieving a good neurological outcome. Following the results of this study, the indication for ECMO should be assessed multifactorial in the future.

Therefore, we investigated our study cohort of 345 OoC VA ECMO patients for significant associations in these various areas that were known to us prior to ECMO implantation. In selecting the potential outcome variables, we relied on parameters that are routinely measured in most hospitals, including smaller clinics, and were therefore immediately available for assessing the indication for VA-ECMO. We were able to demonstrate that factors such as BMI, MAP, ScvO2, LDH, platelet count, prothrombin value, D-dimer, and inflammatory parameters like interleukin 8 and TNF-alpha had an impact on neurological and/or functional outcomes prior to OoC VA ECMO implantation.

The impact of BMI on outcomes in critically ill patients has been well-established for some time [35, 36]. Several explanations have been proposed as to why overweight affects outcomes, including the increased intra-abdominal pressure associated with obesity, which can impair ventilation and perfusion [37]. Additionally, obesity has been linked to chronic inflammation, which can be exacerbated by shock with malperfusion and the need for extracorporeal therapies [38]. Interestingly, on the other hand also better survival rates in overweight patients on extracorporeal support have been reported [39]. Although this study focused on VV ECMO, an important takeaway is that obesity should not be considered an absolute contraindication for extracorporeal support. Even though the implementation of an extracorporeal procedure, from initiation to weaning, is significantly more challenging and complicated in overweight patients [40]. It has long been known that a reduced MAP is associated with poor neurological outcomes, and this has been further confirmed in recent studies on ECPR for VA ECMO patients [41, 42]. Insufficient perfusion pressure, associated with tissue malperfusion and increased tissue damage, as indicated by elevated LDH levels, has also been demonstrated in other organs in prospective studies [43]. Especially in the case of OoC VA ECMO implantation, the goal should be to achieve rapid initiation of the device. Until adequate tissue perfusion is achieved through ECMO, a MAP of > 54 mmHg should be targeted. In cases where classical catecholamine therapy fails, decatecholaminization using methylene blue can be considered as an alternative [44].

In 2001, the international society of thrombosis and haemostasis (ISTH) defined disseminated intravascular coagulation (DIC) as an acquired syndrome characterized by intravascular coagulation activation and loss of localization, resulting from various causes that can damage the microvasculature and, if severe enough, lead to organ dysfunction [45]. Current evidence supports the notion that DIC is also triggered by acute systemic inflammatory response resulting in endothelial dysfunction cause by hypoxemia and malperfusion in shock [46]. It is therefore not surprising that we observed a higher degree of DIC, with elevated D-dimer levels, decreased platelet count, and increased INR/prothrombin time, in the groups with poor outcomes.

However, since it was suspected that the individual influencing factors may not all carry the same level of importance, a SHAP importance analysis was conducted.

For mid-term and long-term (functional and neuronal) outcome, the MAP was identified as the most important variable. This observation aligns with the findings of colleagues who investigated MAP as an outcome parameter for ECLS [42]. Patients with better hemodynamic stability and coagulation profiles had significantly improved chances of survival with favorable neurological and functional outcomes.

After the arrangement and linkage of the individual outcome variables to decision trees, we were able to demonstrate that multi-organ failure measured by SOFA-score was also predictor of poor outcomes. This finding aligns closely with the results published by Nicholas et al. in 2019. In their study, the authors demonstrated that mortality is significantly increased in cases of multiple organ dysfunction syndrome (MODS), which is triggered by an uncontrolled inflammatory response in the body [47]. Similarly, in our study, we were able to show that an increased inflammatory response (Interleukin – 6; Interleukin—8) is associated with a worse outcome.

### Strengths and limitations of this study

With 345 patients/cases for the mid-term outcome and 284 patients/cases for the long-term outcome, to our knowledge this study currently represents the largest dataset on out-of-center implanted VA ECMO. Since we aimed to include only standard parameters that are routinely available in almost every ICU through a single measurement, it should be feasible to apply this approach to a large number of patients.

But there are also several limitations to this study that should be noted. In contrast to the other mortality studies on VA ECMO mentioned above, which were prospectively designed and randomized controlled trials, our study is a purely retrospective, single-center investigation [32]. The retrospective nature of the study is reflected, for example, in the fact that not all measurements were available for every patient, and these missing data could not be retrospectively reassessed. For example: since specific inflammatory parameters (such as TNF-alpha or interleukins) are not routinely measured in every hospital, the authors do not generally recommend the standard inclusion of these values in the decision-making process for ECMO indication. In this study, inflammatory parameters were available in approximately 70% of cases prior to ECMO implantation. However, since inflammation, as represented by its surrogate parameters, can be indicative of tissue damage and has prognostic implications, the authors believe that inflammation should not be entirely excluded from the decision-making process for ECMO indication.

Another limitation is the loss of 61 patients in the long-term outcome group during the follow-up period. These patients could have potentially influenced the outcome or the significance of certain variables, and their absence remains an unrecognized factor.

This study is subject to selection bias. Patients included in the study were those deemed eligible for ECMO therapy by two independent ECMO physicians. As such, randomization was not performed. This may account for the observed functional outcomes, which appear to be comparable to overall survival rates reported in other studies [15]. Additionally, the study observed that the distance from the implantation site to our center was greater in the group of CPC 1 patients, which might suggest that ECMO therapy for these patients was likely initiated only when particularly favorable outcomes were anticipated.

A favorable functional long-term outcome is the goal of any therapy. We intentionally chose to focus on this aspect in our study. The functional outcome, measured using the Karnofsky or ECOG scales, as applied in our work, historically originates from oncology and is used to characterize the status of patients with chronic diseases [48]. In the treatment of cardiocirculatory acute conditions (e.g., resuscitation, ECMO therapy), the CPC score has become established as a measure of outcome, particularly due to the ECLS studies [26, 49]. To ensure comparability with as many existing ECMO studies as possible, while also examining the functional long-term outcome of interest to us, we decided to use both the CPC score and the ECOG scale, publishing both sets of results in parallel in this study. However, using two different outcome measurement tools at different time points presents challenges, as it necessitated separate analysis of each outcome variable. A direct comparison between the two outcome measures is not always feasible. Unfortunately, in the follow-up for functional outcome (ECOG), the CPC score was inconsistently documented, preventing a comparative analysis of CPC and ECOG in this study. Due to the differing scales, it is possible that a patient who would receive a CPC score of 1, indicating a good outcome according to our definition, might be classified as having a worse outcome in the ECOG system with a score of 2 [27]. Another aspect that influences the comparability of the two measurement instruments in relation to the outcome is the presence of intracranial pathologies (stroke or hemorrhage). In 13.2% of our cases, at least one of these conditions was identifiable. While the CPC scale indicated a better neurological prognosis, a larger proportion of patients were found to have more severe impairments in the functional assessment. It appears that the ECOG scale is associated with a higher discriminative ability for the actual outcome in cases involving intracranial pathology.

A reduction of decision trees to a simple"yes"or"no"may initially seem less confusing and more straightforward for clinical practice. However, from our perspective and experience, this does not reflect the reality of patients with such complex conditions. Of course, it would be desirable to develop a simple"yes"or"no"tool. However, the indication for such a highly invasive procedure as VAECMO therapy requires a much more differentiated decision-making basis.

## Conclusion

Setting sensible indications and establishing a thoughtful decision-making process for urgent OoC VA ECMO implantation is challenging but essential for achieving a good outcome. Long-term survival with good neurological and functional outcomes in patients with cardiopulmonary failure treated with OoC VA ECMO is comparable to that of other VA ECMO patients who are treated in-center from the outset. The outcome following OoC VA ECMO therapy is multifactorial and requires the consideration of more than one variable to provide an adequate prognostic assessment. This study identified several potential outcome variables in the out-of-center VA ECMO implantation setting, with each variable differing in its significance for the outcome. By linking these variables, a more valid prognostic assessment can be made prior to VA ECMO implantation in the out-of-center setting. The use of standard parameters for prognostic assessment enables a quick determination of the indication in daily clinical practice. Among the variables studied, MAP holds the greatest importance. The more organ systems are affected by hypoperfusion, the lower the chances of achieving a favorable neurological or functional outcome. Measuring long-term outcomes using scoring systems for functional outcomes is feasible but more complex and associated with a higher number of missing data points. Further prospective multicenter studies using the new prognostic decision tool are necessary to validate the results of this study.

## Supplementary Information

Supplementary Material 1.

Supplementary Material 2.

Supplementary Material 3.

## Data Availability

No datasets were generated or analysed during the current study.

## Abbreviations

BMI: Body Mass Index
cm: Centimeter
CPC: Cerebral Performance Category
CRP: C-Reactive Protein
D-Dimer: Fibrin Split Product
E.g.: For example
ECLS: Extracorporeal Life Support
ECMO: Extracorporeal Membrane Oxygenation
ECOG: Eastern Cooperative Oncology Group
ELSO: Extracorporeal Life Support Organization
GOT: Glutamate Oxaloacetate Transaminase
Hb: Hemoglobin
IL—2: Interleukin 2
IL—6: Interleukin 6
IL—8: Interleukin 8
INR: International Normalized Ratio
INVOS: Near-Infrared Spectroscopy (NIRS)
ISTH: International Society of Thrombosis and Haemostasis
kg: Kilogram
km: Kilometer
L: Liter
LCO: Low Cardiac Output
LCO-ongCPR: Low Cardiac Output with Ongoing Cardiopulmonary Resuscitation
LCO-interCPR: Peri-Interventional Cardiopulmonary Resuscitation
LCO-postECB: Post-Cardiotomy Failure after Cardiopulmonary Bypass
LCO-preCPR: Low Cardiac Output after Successful Resuscitation with Return of Spontaneous Circulation
LDH: Lactate Dehydrogenase
m^2^: Square Meter
MAP: Mean Arterial Pressure
mg: Milligram
min: Minute
mL: Milliliter
mmHg: Millimeters of Mercury
OoC: Out of Center
OoC VA ECMO: Out of Center Venoarterial ECMO
Pg: Picogram
PTT: Partial Thromboplastin Time
ScVO₂: Central Venous Oxygen Saturation
Sec: Seconds
SHAP: SHapley Additive exPlanations
SOFA-Score: Sequential Organ Failure Assessment Score
TNFα: Tumor Necrosis Factor Alpha
VA ECMO: Veno arterial ECMO
μg: Microgram
μL: Microliter
