# Supplementary material for: Long-term outcomes of out-of-center veno-arterial ECMO cannulation for cardiopulmonary failure: investigation of prognostic parameters for a decision support tool – a 16-year retrospective study
Source: Scand J Trauma Resusc Emerg Med. 2025 May 12;33:81. doi: 10.1186/s13049-025-01401-7 (PMC12070683; doi:10.1186/s13049-025-01401-7)
Supplement: Supplementary file 1 — Supplementary Material 1. [file 13049_2025_1401_MOESM1_ESM.pdf]

Abfragebogen für Patienten mit ECMO unterstütztem Intensivtransport

Internistische Int. St. 93: Tel 0941- 944 1 7350 Fax 944 7365
Herzchirurgie St. 97: Tel 0941-944 9834 Fax 944 9832 | Anästhesiologische Int. St. 90: 0941- 944 7920 Fax 944 7924

**Externe Klinikdaten**		**Patientendaten**	
**Datum:**	**Uhrzeit:**	**Patientenname:**	
**Klinik:**		**Geburtsdatum:**	
**Station:**		**Größe:**	**Gewicht:**
**Anrufer:**		**Aufnahmedatum:**	
**Rückrufnummer:**			

**Anamnese**		
**Keimnachweis:**	**Abstriche:**	**Multiresistente Erreger:**
**Antibiosen:**		**Isolationspflichtig:** ☐ ja ☐ nein
**Neurologie:**		**CT vorhanden:** ☐ ja ☐ nein

**Respiration**			**Hämodynamik**
**Beatmungsbeginn:**			**Noradrenalin (mg/h):**
**FiO_2:**		**pH:**	**Adrenalin (mg/h):**
**AMV:**	**TV:**	**P_aO_2**	**Blutdruck:**
**PEEP:**	**PIP:**	**P_aCO_2:**	**ZVD:**

**Labor**			
**Kreatinin:**	**Bilirubin:**	**Laktat:**	**CVVHF:** ☐ ja ☐ nein
**GOT:**	**Hb:**		**CT-Thorax:**
**PTT:**	**Leukozyten:**		**ECHO/TEE:**
**INR:**	**Thrombozyten:**		**IABP:**

**Zugänge**		
**ZVK:**	**Ort:**	**Shaldon:**
**Arterie:**	**Ort:**	
**Gefäßprothesen in Leiste:**		
**Thoraxdrainagen:**		

**Informationen an** (Funk 1485)	
**Kardiotechniker:**	**HTC OA:** (Bei kardialem Versagen):

**Transport mit ECMO**	☐ ja ☐ nein	
**Organversagen primär:**	☐ kardial ☐ respiratorisch	
**Aufnehmende Station im UKR:**		
**Übernahme zugesagt:**	☐ ja ☐ nein	**Uhrzeit:**
**Bearbeiter:**		
