# Supplementary material for: Long-term outcomes of out-of-center veno-arterial ECMO cannulation for cardiopulmonary failure: investigation of prognostic parameters for a decision support tool – a 16-year retrospective study
Source: Scand J Trauma Resusc Emerg Med. 2025 May 12;33:81. doi: 10.1186/s13049-025-01401-7 (PMC12070683; doi:10.1186/s13049-025-01401-7)
Supplement: Supplementary file 2 — Supplementary Material 2. [file 13049_2025_1401_MOESM2_ESM.docx]

**Figures legends**

**Figure 1: Flowchart of patient selection**:

***Figure 1:*** *Flowchart of exclusion criteria for OoC VA ECMO patients from 2006 to 2022.*

**Figure 2: SHAP Importance: The size of variable influence on the CPC outcome in out-of-center VA ECMO patients.**

***Figure 2****: SHAP Importance: The size of the variable influence, sorted in descending order of importance, on the CPC outcome, expressed as a percentage.*

**Figure 3: SHAP Importance: The size of variable influence on the ECOG outcome in out-of-center VA ECMO patients.**

**Figure 3**: SHAP Importance: The size of the variable influence, sorted in descending order of importance, on the ECOG outcome, expressed as a percentage.

# **Figure 4: Decision tree mid-term outcome (CPC) in OoC VA ECMO**

***Figure 4:*** *The outcome variables are presented in descending order of importance, organized in a decision tree format. For each variable (represented as nodes), the calculated threshold, sample size (N), percentage distribution (CPC group and total population), and p-value are provided. The significance level was set at p < 0.05.*

# **Figure 5: Decision tree long-term outcome (ECOG) in OoC VA ECMO**

# ***Figure 5:*** *The outcome variables are presented in descending order of importance, organized in a decision tree format. For each variable (represented as nodes), the calculated threshold, sample size (N), percentage distribution (ECOG group and total population), and p-value are provided. The significance level was set at p < 0.05.*

**Tables legends**

**Table 1: Demographic data in cerebral performance category (CPC) outcome group**

***Table 1:*** *Demographic data for CPC in out-of-center VA ECMO patients between 2006 and 2022. The medians (25th percentile / 75th percentile) are reported. The significance level was set at p ≤ 0.05. The significance level was set* *at p ≤ 0.05, and significant values are marked with an asterisk (*) in the table.*

**Table 2: Demographic data in ECOG performance scale outcome group**

***Table 2:*** *Demographic data for ECOG in out-of-center VA ECMO patients between 2006 and 2022. The medians (25th percentile / 75th percentile) are reported. The significance level was set at p ≤ 0.05. The significance level was set* *at p ≤ 0.05, and significant values are marked with an asterisk (*) in the table.*

**Table 3: Cause of cardiopulmonary failure in OoC VA ECMO**

***Table 3:*** *Classification of patients according to underlying cause of cardiopulmonary failure, with patient count and percentage of the total population. Entries depict the number of patients (percentage).*

**Table 4: Regensburger indication code for OoC VA ECMO**

***Table 4:*** *OoC VA ECMO indications according to the coding system used at the University Hospital Regensburg. Entries depict the number of patients (percentage)*

**Table 5: Cerebral performance category (CPC) outcome in OoC VA ECMO**

***Table 5:*** *Outcome in CPC group in the OoC VA ECMO population; Entries represent the number of patients (percentage)*

**Table 6: ECOG performance scale outcome in OoC VA ECMO**

***Table 6:*** *Absolute number and percentage of each ECOG performance scale outcome group in the OoC VA ECMO population; Entries represent the number of patients (percentage)*

**Table 7: Disease dynamic and organ failure prior to OoC VA ECMO CPC outcome**

***Table 7:*** *Presentation of disease severity due to perfusion disturbance with evidence of organ failures. Specifically, renal failure and the SOFA score, commonly used in sepsis to assess organ failures, are highlighted. Disease dynamics are scaled by the duration of ventilation prior to ECMO, as well as the time from the onset of illness to the need for ECMO therapy. Values were presented in number of patients (percentage) and main (QRS values) (25th and 75th percentiles). The significance level was set* *at p ≤ 0.05, and significant values are marked with an asterisk (*) in the table.*

**Table 8: Coagulation parameters (platelets, PTT, Quick, INR, D-Dimer)**

***Table 8:*** *Comparison of cellular and plasma coagulation in OoC VA ECMO patients with good and poor outcomes. The upper part of the table presents CPC outcomes, while the lower part shows ECOG outcomes. Values were presented as main values and QRS values (25th and 75th percentiles).* *The significance level was set at p ≤ 0.05, and significant values are marked with an asterisk (*) in the table.*

**Table 9: Inflammatory markers prior to OoC VA ECMO**

***Table 9****: Comparison of inflammatory markers in out-of-center (OoC) VA ECMO patients with good and poor outcomes. The upper part of the table presents CPC outcomes, while the lower part shows ECOG outcomes. Values were presented as main values and QRS values (25th and 75th percentiles).The significance level was set at p ≤ 0.05, with significant values marked by an asterisk (*) in the table.*
