# Supplementary material for: Long-term outcomes of out-of-center veno-arterial ECMO cannulation for cardiopulmonary failure: investigation of prognostic parameters for a decision support tool – a 16-year retrospective study
Source: Scand J Trauma Resusc Emerg Med. 2025 May 12;33:81. doi: 10.1186/s13049-025-01401-7 (PMC12070683; doi:10.1186/s13049-025-01401-7)
Supplement: Supplementary file 3 — Supplementary Material 3. [file 13049_2025_1401_MOESM3_ESM.docx]

Supplement:

The following table contains the most common complications in the OoC setting of VA ECMO implantation from 2006 to 2022, including the percentage of distal perfusions performed (percentage of the total number).

|  | **N = 345** |
| --- | --- |
| **Overall Complication** | **88 of 345** (25.5%) |
| **Ischemia of the cannulated leg** | **28 of 345** (8,1%) |
| **Vascular injury incl Retroperitoneal hematoma** | **38 of 345** (11.0%) |
| **Bleeding at the cannulation site** | **22 of 345** (6,4%) |
| **abdominal compartment syndrome** | **21 of 345** (6,1%) |
| **Distale Antegrade Perfusion** | **162 of 345** (47,0%) |
